# Laparoscopic diagnostic peritoneal lavage (L-DPL): A method for evaluation of penetrating abdominal stab wounds

**DOI:** 10.1186/1749-7922-1-3

**Published:** 2006-03-24

**Authors:** Michael M Krausz, Benyamine Abbou, Dan D Hershko, Ahmad Mahajna, Daniel S Duek, Bishara Bishara, Shlomo H Israelit

**Affiliations:** 1 Department of Surgery A', Rambam Medical Center, and The Bruce Rappaport Faculty of Medicine, Technion-Israel Institute of Technology, Haifa, Israel

## Abstract

**Background:**

The management of penetrating abdominal stab wounds has been the subject of continued reappraisal and controversy. In the present study a novel method which combines the use of diagnostic laparoscopy and DPL, termed laparoscopic diagnostic peritoneal lavage (L-DPL) is described

**Method:**

Five trauma patients with penetrating injuries to the lower chest or abdomen were included. Standard videoscopic equipment is utilized for the laparoscopic trauma evaluation of the injured patient. When no significant injury is detected, the videoscope is withdrawn and 1000 mL of normal saline is infused through the abdominal trochar into the peritoneal cavity, and the effluent fluid studied for RBCs, WBC, amylase debry, bile as it is uced in regular diagnostic peritoneal lavage

**Results:**

Laparoscopic peritoneal lavage (L-DPL) was then performed and proved to be negative in all 5 patients. RBC lavage counts above 100,000/mcrl were not considered as a positive lavage result, because the bleeding source was directly observed and controlled laparoscopically. All patients recovered uneventfully and were released within 3 days. This procedure combines the visual advantages of laparoscopy together with the sensitivity and specificty of DPL for the diagnosis of significant penetrating intra-abdominal injury, when the diagnostic strategy of selective consevatism for abdominal stab wounds is adopted.

**Conclusion:**

A method of laparoscopic diagnostic peritoneal lavage (L-DPL) in hemodynamically stable patients with penetrating lower thoracic or abdominal stab wounds is described. The method is especially applicable for trauma surgeons with only basic experience in laparoscopic technique. This procedure is used to obtain conclusive evidence of significant intra-abdominal injury, confirm peritoneal penetration, control intra-abdominal bleeding, and repair lacerations to the diaphragm and abdominal wall. The combination of laparoscopy and DPL afforded by the L-DPL method adds to the sensitivity and specificity of DPL, and avoids under or over sesitivty, that have limited the use of DPL in the hemodynamically stable trauma patients with suspicious or proven peritoneal penetration.

## Background

The management of penetrating abdominal stab wounds has been the subject of continued reappraisal and controversy. Traditionally, the concern for delayed diagnosis of intra-abdominal injuries has led many trauma centers to advocate mandatory abdominal exploration whenever a stab wound was proven or suspected to have penetrated into the abdominal cavity [1,2]. This liberal approach resulted in a reluctant acceptance of a 50% incidence of non-therapeutic laparotomies in an attempt to prevent delayed diagnosis of intra-abdominal injuries [3].

Selective operative management of asymptomatic patients ("selective conservatism") was therefore advocated in 1960 when it was recognized that 25% to 33% of the patients with stab wounds have no peritoneal penetration, and of those with proven penetration, significant abdominal injuries are present only in about 45% [4,5].

In order to overcome the diagnostic delay and reduce the number of non-therapeutic laparotomies, diagnostic peritoneal lavage (DPL) was applied which resulted in reduced incidence of non-therapeutic celiotomies of as little as 7%–15% [6].

Diagnostic laparoscopy in trauma patiens has been first described by Gazzaniga in 1976. Despite this long history of sporadic use, indications for its use in trauma casualties remain controversial. This technique has primarily been used to detect peritoneal penetration in tangential wounds of the abdominal wall and for evaluation of the diaphragm in patients with lower thoracic wounds [7]. More extensive laparoscopic examination of the stomach, colon and small bowel and repair of diaphragmatic and hollow viscus injuries is performed only by a few trauma surgeons skilled in advanced laparoscopic techniques. The main concern even in the hands of the experienced surgeon in advanced laparoscopic techniques is that small lacerations of the bowel may be overlooked.

In the present study a novel method which combines the use of diagnostic laparoscopy and DPL, termed laparoscopic diagnostic peritoneal lavage (L-DPL) is described. This method was used for evaluation of significant intra-abdominal injuries in hemodynamically stable trauma patients with penetrating lower thoracic or abdominal stab wounds. We believe that this procedure can be safely performed by the trauma surgeon with basic skills in the laparoscopic technique.

## Method

Five trauma patients with penetrating injuries to the lower chest or abdomen were included.

The entrance criteria for diagnostic laparoscopy were as follows:

a. The patient was hemodynamically stable, with negative or equivocal abdominal physical findings, b. Stab wound in the lower chest (nipple to costal margin), c. Pentration into the abdominal cavity proven by wound exploration, d. Inconclusive wound exploration, e. suspected peritoneal penetration on tripple-contrast abdominal CT scan or FAST in stab wounds to the flank or back, and f. No significant intra-abdominal injury that necesitates open laparotomy was detected by abdominal CT or FAST.

### Technique of Laparoscopic Diagnostic Peritoneal Lavage (L-DPL)

Standard videoscopic equipment is utilized for the laparoscopic trauma evaluation of the injured patient. In the hemodynamically stable patient, the procedure starts with local wound exploration (LWE) to determine peritoneal penetration (Table). This is sometimes difficult and tedious especially in obese patients or patients with thick abdominal musculature. If no penetration is proven the patient is observed and discharged without further investigations. When penetration is proven or exploration of the wound is equivocal, FAST and/or triple-contrast CT can be added to demonstrate the presence of local signs of peritoneal penetration, intra-abdominal bleeding, and intra-abdominal injuries. A nasogastric tube and a urinary bladder catheter are inserted for decompression prior to performing laparoscopy. A 30 degree angled 10 mm scope is preferred, as this provides optimal visualization of difficult areas such as the posterior aspect of the diaphragm and anterior abdominal wall. Initial insufflation of CO_2 _is conducted with either a Verres needle or a Hassan cannula. Initial insufflation pressures in the trauma setting should be limited to 8–10 mmHg in the patient with penetrating thoraco-abdominal wounds as this will minimize the development of tension pneumothorax in patients with diaphragmatic defects. If no diaphragmatic injury is detected in lower thoracic stab wounds (between the nipple and costal margin), the procedure is terminated without lavage. When a diaphragmatic tear is detected, it can be repaired laparoscopically by non-absorbable sutures before peritoneal lavage is performed.

In abdominal stab wounds, after exclusion of diaphragmatic injury, insufflation pressure can be increased to 15 mmHg, as used in standard laparoscopy. One or more ports can be added for laparoscopic repair, or if improved exposure is needed. When significant hemorrahage cannot be controlled laparoscopically, or enteric contents are encountered during initial laparoscopic assessment, the examination is terminated and converted to an open exploratory laparotomy.

When no significant injury is detected, the videoscope is withdrawn and 1000 mL of normal saline is infused through the abdominal trochar into the peritoneal cavity. The patient is rolled from side to side to promote mixing of the lavage fluid with the peritoneal contents. Fluid returning through the trochar after mixing is inspected and immediately sent for quantitative analysis. The lavage fluid is considered positive if feces, bile, food, bacteria on Gram stain, >500 white blood cells/mcrl, or increased amylase levels are detcted. A red blood cell count >100,000/mcrl by itself is not considered positive.

If the lavage fluid return proves to be positive, the procedure is immediately converted to an open exploratory laparotomy. When the lavage fluid proves to be negative, a J-P closed drain is left in the peritoneal cavity, the procedure is terminated without open exploration. Postoperatively the patient is carefully observed for 24 hours, after which the J-P drain is extracted, and the patient can be discharged. In case of change in the patient' s condition or suspicious fluid return appears in the drain during observation, the lavage procedure can be repeated. Complete intraoperative evaluation of the lavage fluid return was accomlished in less than 30 minutes.

**Figure 1:**
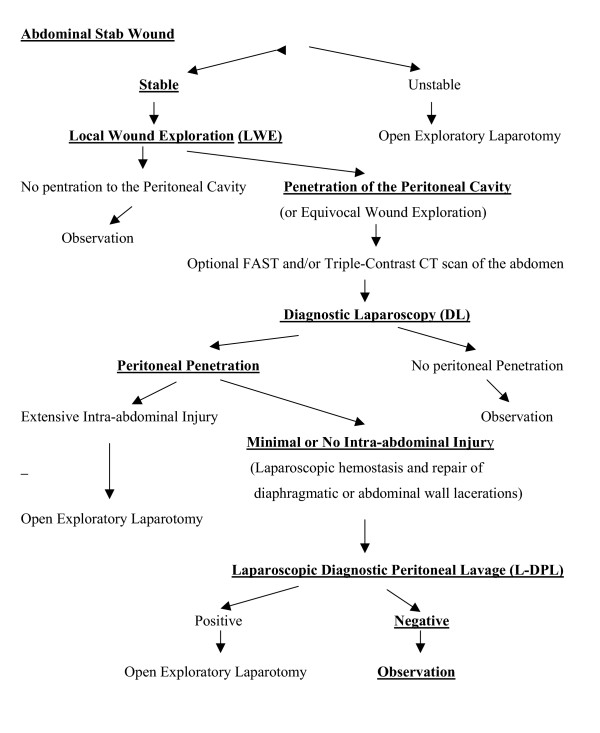
Algorithm for Treatment of Abdominal Stab Wounds by Laparoscopic Diagnostic Peritoneal Lavage (L-DPL).

## Results

There were 2 lower thoracic stab wounds, 2 abdominal stab wounds and one stab wound at the costal margin. When peritoneal penetration was suspected or proven, a thorough diagnostic laparoscopic survey was performed, actively bleeding vessels were coagulated, and diaphragmatic or abdominal wall lacerations repaired laparoscopically. Laparoscopic peritoneal lavage (L-DPL) was then performed and proved to be negative in all 5 patients.

RBC lavage counts above 100,000/mcrl were not considered as a positive lavage result, because the bleeding source was directly observed and controlled laparoscopically. All patients recovered uneventfully and were released within 3 days. In no patient was repeated peritoneal lavage necessary, and the J-P drains were removed on POD 1.

## Discussion

A procedure of combined diagnostic laparoscopy and laparoscopic diagnostic peritoneal lavage (D-DPL) for evaluation of hemodynamically stable trauma patients, with lower thoracic or anterior abdominal stab wounds, that have penetrated the abdominal cavity, is hereby described. This procedure combines the visual advantages of laparoscopy together with the sensitivity and specificty of DPL for the diagnosis of significant penetrating intra- abdominal injury, when the diagnostic strategy of selective consevatism for abdominal stab wounds is adopted. An important advantage of the procedure is that it can be safely performed by trauma surgeons with only basic experience in the laparoscopic technique.

The accuracy of DPL has been reported to be greater than 95% and has proven to be highly reproducible among centers [6], but oversensitivity of this procedure remains a major problem. DPL has proved particularly unreliable in detecting injuries to diaphragm, regardless of cell count used to mandate laparotomy for penetrating trauma. Diaphragmatic tears have been observed with low red cell counts of 1600/mcrl [8]. Reducing the citeria for a positive DPL finding to a red cell count as low as 1000/mcrl [9] would significantly reduce the the false negative rate of DPL, but would correspondingly increase the negative and nontherapeutic laparotomy rate [9].

Diagnostic laparoscopy is commonly used only to detect injury to the diaphragm with penentration into the abdominal cavity. Once peritoneal penetration has been detected, the laparoscopic procedure is usually terminated and converted to an open procedure, because most trauma surgeons with only basic experience in laparoscopic technique, regarded laparoscopic abdominal exploration unsafe because it could not definitely exclude small but significant intra-abdominal injury, such as a small intestinal perforation.

Salvino et al. [8] suggested the use diagnostic laparoscopy as a screening procedure for the need of laparotomy in patients where DPL was proven positive or indeterminate, regardless of the DPL criteria that define a "positive" result, in order to reduce unnecessary laparotomies. It was estimated that almost a third of patients with positive DPL results would potentially benefit from non-surgical management based on diagnostic laparoscopy evaluation of intra-abdominal injuries. The use of DPL as the sole criterion for diagnostic laproscopy will miss a significant number of penetrating injuries to the diaphragm, indeterminate lavage results, and retroperitoneal injuries.

DeMaria et al. [10] compared mandatory celiotomy to laparoscopy in hemodynamically stable patients with thoraco-abdominal stab wounds. Non-therapeutic celiotomy was significantly less common in the group initially evaluated by laparoscopy (19% Vs 57%). The sesitivity, specificity and accuracy of laparoscopic evaluation was also superior when compared to DPL in predicting the need for therapeutic intervention at open abdominal exploration.

Our method of laparoscopic diagnostic peritoneal lavage (L-DPL) in hemodynamically stable patients is indicated not only for stab wounds in the lower thoracic region but particularly for evaluation of abdominal stab wounds with proven or suspcious pritoneal penetration. Laparoscopic evaluation allows more specific diagnosis of the nature and degree of injury as well as the need for laparotomy. Once peritoneal penetration through the abdominal wall or diaphragm is proven, the injury in the abdominal wall can or diaphragm can be repaired laparoscopically, and diagnostic peritoneal lavage is performed through the abdominal trochar before termination of the surgical procedure. Reliance on the findings of laparoscopy avoided in our study a nontherapeutic laparotomy, even when a "DPL positive" RBC count of more than 100,000/mcrl was detected

A disadvantages of L-DPL are that it is an invasive procedure, that is usually performed under general anesthesia. There are potential dangers of tension pneumothorax and gas embolization in trauma patient [2,11]. Compulsive monitoring of trauma patients undergoing the L-DPL procedure with graded insufflation of gas into the peritoneal cavity, is mandatory in order to prevent these life threatening complications.

L-DPL is also recommended for trauma surgeons familiar with advanced laparoscopic techniques, when no significant injury was detected by a thorough laparoscopic survey. When laparoscopic diagnostic and therapeutic procedures, such as hemostasis of liver or spleen lacerations, suture repair of the diaphragm, or thorough examination of the small and large bowel have been accomplished, the addition of L-DPL before termination of the procedure, increases the confidence that a small but significant injury, such as a small bowel perforation was not overlooked.

The advantages in diagnosis and therapeutics afforded by the L-DPL method are also expected to reduce morbidity, hospital costs, and length of hospitalization associated with non-therapeutic open laparotomies in patients with penetrating lower thoracic or abdominal injuries.

## Conclusion

A method of laparoscopic diagnostic peritoneal lavage (L-DPL) in hemodynamically stable patients with penetrating lower thoracic or abdominal stab wounds is described. The method is especially applicable for trauma surgeons with only basic experience in laparoscopic technique. This procedure is used to obtain conclusive evidence of significant intra-abdominal injury, confirm peritoneal penetration, control intra-abdominal bleeding, and repair lacerations to the diaphragm and abdominal wall. The combination of laparoscopy and DPL afforded by the L-DPL method adds to the sensitivity and specificity of DPL, and avoids under or over sesitivty, that have limited the use of DPL in the hemodynamically stable trauma patients with suspicious or proven peritoneal penetration.

## Abbreviatios

L-DPL- Laparoscopic peritoneal lavage

CT- Computed tomography

FAST- Focused abdominal sonography for trauma

LWE- Local wound exploration

## Authors' contributions

Michael M. Krausz, M.D – Has made substantial contributions to conception, design, collection, and analysis of data

Benyamine Abbou, M.D – Has made substantial contributions to design and conception

Dan D. Hershko, M.D – Has made substantial contributions in revising critically the Manuscript

Ahmad Mahajna MD – Has made substantial contributions in revising critically the

Manuscript

Daniel S. Duek, M.D – Has made substantial contributions in collecting data

Bishara Bishara M.D – Has made substantial contributions in collecting data

Shlomo H.Israelit, M.D. Has made substantial contributions in design and analysis of data
